# *“Being You. A New Science of Consciousness”* by Anil Seth

**DOI:** 10.17505/jpor.2026.29051

**Published:** 2026-03-26

**Authors:** Lars-Gunnar Lundh

**Affiliations:** Department of Psychology, Lund University, Sweden

**Keywords:** consciousness, perception, feeling, self, predictive processing, affordances, perceptual systems, active inference, Anil Seth, Gibson, Wittgenstein

## Abstract

In this book, Anil Seth advocates a predictive processing approach to the understanding of conscious experience, which he claims would amount to something like a Copernican revolution. In three separate parts of the book, Seth discusses conscious *level*, conscious *content*, and conscious *self*. Among the strengths of the book is an ambition to stay close to the rich phenomenology of conscious experiences. The phenomenological openness, however, is counteracted by an ambition to reduce all aspects of consciousness to predictive processing.

The neuroscientist Anil Seth starts his book *Being you* (Seth, 2021) with a fascinating thought experiment. It goes like this: Suppose you are offered the deal of having your brain replaced “with a machine that is its equal in every way, so that from the outside, nobody could tell the difference (p. 3). Suppose further that one of the many advantages in favour of this deal is that this new machine is immune to decay and may perhaps even allow you to live forever. At the same time, however, the scientist who provides the offer *cannot* guarantee

that you will have any conscious experiences at all, should you take up this offer. … I suspect you wouldn’t take the deal. Without consciousness, it may hardly matter whether you live for another five years or another five hundred. (Seth, 2021, p. 3-4)

This thought experiment reminds us that conscious experience is of immeasurable value for us, and that this can motivate us to reject the deal that is offered. As Seth (2021) summarizes it:

For each of us, our conscious experience is all there is. Without it there is nothing at all: no world, no self, no interior and no exterior” (p. 3)

This strikes me as a beautiful and convincing example of how philosophical investigations, although they can never lead to the discovery of strictly new facts, can provide us with *a better understanding of what we already know*. As Wittgenstein put it,

The problems are solved, not by giving new information, but by arranging what we have always known. (Wittgenstein, 1953, §109).

Philosophical investigations, according to Wittgenstein, often means to *remind* ourselves of things we already know:

The work of the philosopher consists in assembling reminders for a particular purpose. (Wittgenstein, 1953, §127).

The important thing about Seth’s thought experiment is that it reminds us of how immeasurably important consciousness is for each of us. This may be seen in contrast to the almost total lack of interest researchers in psychological science showed for consciousness during the 20^th^ century. Earlier 19^th^ century psychologists, such as William James (1890), were quite interested in understanding the nature of consciousness. But during the 20^th^ century, the field was dominated by behaviourism and information processing approaches, which largely ignored questions about consciousness. Now, well into 21^th^ century, we see a revival of interest in questions about the nature of consciousness both among philosophers, psychologists, and neuroscientists.

Seth’s ambition is not only to remind us of the immeasurable personal importance of consciousness, but he also wants us to see consciousness in a new way, in what amounts to something like a Copernican revolution. And here again, Wittgenstein is relevant. On page 79, Seth quotes the following philosophical dialogue between Wittgenstein and Elizabeth Anscombe:

Ludwig Wittgenstein: “Why do people say that it was natural to think that the sun went around the Earth rather than that the Earth turned on its axis?”

Elizabeth Anscombe: “I suppose, it looked as if the sun went round the Earth.”

Ludwig Wittgenstein: “Well, what would it have looked like if it had *looked* as if the Earth turned on its axis?”

Here Wittgenstein reminds us of the fact that we always see things from the perspective of our own body. As the embodied Earth-bound creatures we are, it will always *look* as if the sun moves around the Earth. Yet, we have an ability to distance ourselves *theoretically* from our embodied perspective on the world and arrive at new insights about reality.

When Seth develops his theory about perception as “controlled hallucination”, his ambition is to provide us with a new theoretical perspective on consciousness which amounts to something like a Copernican revolution. Although I am not convinced by his argumentation, I still think his arguments are worth some consideration.

Seth differentiates between three different aspects of consciousness: (1) conscious level; (2) conscious content; and (3) conscious self. In this review I will focus on the two latter, but I will start with a short summary of his chapters about conscious level.

## Conscious Level

Already in the prologue, on page 1 of his book, Seth raises questions about levels of consciousness. This is done in relation to anaesthesia during surgery:

General anaesthesia is very different from sleep. It has to be: if you were asleep, the surgeon’s knife would quickly wake you up… Under profound anaesthesia, the brain’s electrical activity is almost entirely quietened – something that never happens in normal life, awake or asleep. (Seth, 2021, p. 1)

The differentiation between deep anesthesia, sleep, and ordinary conscious awareness suggests that may speak about levels of consciousness, and perhaps even develop a *measure* of degree of consciousness. In the first part of the book, Seth reviews various attempts to develop such measures, from Massimi’s *perturbational complexity index* (PCI) to *phi* in Tononi’s integrative information theory (IIT).

As Seth describes it, the meaningfulness of these attempts rests on the assumption that consciousness can be seen in analogy with temperature. Temperature is measured by thermometers, and research has shown that temperature is identical with mean molecular kinetic energy. Could it be that consciousness is similarly *identical* to some pattern of processes in the brain (e.g., neural complexity, or causal density)? If so, maybe consciousness could be measured by measuring these neural patterns? Seth, however, ends up seeing consciousness more in analogy with *life* than with temperature

Is consciousness more like *temperature* – reducible to and identifiable with a basic property of the physical (or informational) universe? Or is it more like *life*, a constellation of many different properties, each with its own explanation in terms of underlying mechanisms? The approaches to measuring consciousness we’ve met up to now take their cue from the temperature story, but my intuition is that in the end they may fit better with the analogy from life. (Seth, 2021, p. 58).

No one has so far identified a way of measuring the degree of life. So why should we expect to find ways of measuring the degree of consciousness? On the other hand, it may be argued that, although we have no real reason to develop a measure of degree of life, we *do* have a motive to develop a measure of level of consciousness:

There is an urgent need for new methods of deciding whether consciousness remains after severe brain damage, when patients are given terrifying diagnoses like the ‘vegetative state’ or the ‘minimally conscious state’. (Seth, 2021, p. 36)

But here I wonder whether there is some conceptual confusion involved. Consider again the analogy with temperature: Essential to temperature is that *optimal* temperature is not synonymous with *maximal* temperature – in fact, maximal temperature is as devastating as minimal temperature. If we should look for a mental analogue to degree of temperature we should perhaps search in the direction of degree of *mental activity,* rather than degree of consciousness? It does not seem too far-fetched to assume that optimal mental activity is quite different from maximal mental activity – too much mental activity, for example, may be associated with mental disorders that involve hyperactivity, inattention, and mania.

## Conscious Content

The purpose of the second part of the book, as Seth formulates it, is to

explore the idea that the brain is a ‘prediction machine’, and that what we see, here, and feel is nothing more than the brain’s ‘best guess’ of the causes of its sensory input. (Seth, 2021, p. 76).

This is the core of the predictive processing theory, as applied to conscious experience, and according to Seth this may lead to a Copernican revolution this area.

In Seth’s theoretical perspective, perception is “controlled hallucination”. He starts by anchoring his theoretical approach historically in the writings of Plato and Kant. He refers to Plato’s Allegory of the Cave, according to which our conscious perceptions are like shadows on the wall – “indirect reflections of hidden causes that we can never directly encounter” (p. 80). And then he turns to Kant’s notion of the “things in themselves” as “a mind-independent reality that will always be inaccessible to human perception, hidden behind a sensory veil” (p. 81).

As far as I can see, Seth presents at least three kinds of arguments concerning our limited perceptual access to the world:

1. our *sensory* access to the physical world is limited;
2. our perceptions are not about the physical world as such, but about what is *useful* to us in the world; and
3. our perceptual repertoire is determined by *generative models*, and the perceptual process involves
  - a continuous comparison of the sensory signals generated by these models with incoming sensory signals, and
  - a continuous updating of the generative models as the result of these comparisons, for the purpose of predictive error minimization.

Of these, only the third argument seems directly relevant to Seth’s theory of perception as “controlled hallucination”, whereas the two first ones are rather about the *embodiment-*dependent nature of perception.

### Limitations in our sensory access to the physical world

Seth writes about limitations in our perceptual access to the physical world due to the construction of our senses:

Our visual system, amazing though it is, only responds to a tiny slice of the full electromagnetic spectrum, nestled in between the lows of infra-red and the highs of ultra-violet. (Seth, 2021, p. 84-85)

This is a kind of embodiment-related limitation that can be illustrated in several ways. For example, due to our specific human embodiment, we also have limitations in the *time scale* of events that we can perceive (i.e., some processes are too fast and others too slow for us to perceive), and in the *size* of the phenomena that are available for direct perception (e.g., elementary particles are too small, whereas galaxies are too large). That is, due to the nature of our embodiment in the world, we have perceptual access only to a “tiny slice” of the spatiotemporal world. But still, what we have access to is a slice *of the physical world*.

### The useful-related nature of the perceptual world

The same seems to apply to Seth’s next argument, which is that

We perceive the world not *as it is*, but *as it is useful for us*. (Seth, 2021, p. 138)

This kind of argument could have been framed in terms of Gibson’s (1979) theory of *affordances*. But although Seth refers to affordances briefly in the third part of his book, he does not integrate the concept of affordances into his discussion of the perceptual world.

In Gibson’s (1979) ecological approach to perception, what animals perceive is not primarily colours, shapes, etc. but what the environment *affords* in terms of *possible interactions*, for good or bad. This includes not only what the organism can do in its environment, but also what the environment can do to the organism (e.g., in terms of dangers). In ecological terms, different species occupy different niches in the environment. The niche is the place of the species in the eco-system – its way of life, i.e., what it feeds on, how it utilizes the environment, etc. In short, a niche “is a set of affordances” (Gibson, 1979, p 128).

For example, what constitutes food for one species does not constitute food for another species. What constitutes a surface to walk and stand on varies from one species to another. Water is such a surface for some insects. But affordances are not only species-specific. Variations in the size of the individual, even within the same species, are also of importance. An obstacle can, as Gibson (1979, p. 36) says, “be defined as an animal-sized object that affords collision and possible injury.” The affordances are different for a child than for an adult: “Knee-high for a child is not the same as knee-high for an adult, so the affordance is relative to the size of the individual” (Gibson, 1979, p. 128).

The concept of affordances clearly illustrates how the perceptual world cannot simply be *equated* with an objective physical world. Still, the world of affordances *is* the physical world, but in its physical *relatedness* to an embodied individual. There is nothing “hallucinatory” about these aspects of the perceptual world.

### The role of generative models in the perceptual process

The third argument is the crucial one. According to Seth, our perceptual repertoire is determined by *generative models* in the brain:

Generative models determine the repertoire of perceivable things. (Seth, 2021, p. 107)

This means that without generative models no perception can take place. This clearly opens up for “hallucinations”, or at least *imagination*, as part of the perceptual process.

How, then, are these generative models to be understood? Does it mean, for example, that to be able to perceive a gorilla we must have a generative model of a gorilla in the brain? And what would that mean? As I understand it (although Seth does not express it in this way), it means that we must have acquired the *concept* of gorilla and be able to *imagine* a gorilla even in its absence. This seems to fit with Seth’s claim that the generative model makes us able to *generate the sensory signals* that would be expected *if* we had a real gorilla in front of us:

In order to perceive a gorilla, my brain needs to be equipped with a generative model capable of generating the relevant sensory signals – the sensory signals that would be expected were a gorilla to be actually present. (Seth, 2021, p. 107)

On this account, if we think we see a gorilla but are not immediately *sure* whether it is a real gorilla, the brain *compares* the incoming sensory signals with the internally generated signals. The generative model is said to provide *perceptual predictions* that are tested against the incoming data to *minimize prediction errors*.

These models provide the flow of perceptual predictions which are compared against incoming sensory data to form prediction errors – which then prompt updated predictions as the brain tries to minimize these errors. (Seth, 2021, p. 107)

In other words, there seems to be a continuity between imagination and perception – both make use of the same sensory areas in the brain. The major difference is that *images* are internally generated (i.e., they are “uncontrolled hallucinations”, in Seth’s terminology), whereas *perceptions* are the result of a comparison between internally and externally generated sensory signals (i.e., “controlled hallucinations”).

During this process, the brain’s predictions are continually updated to minimize prediction errors. In other words, this might be described as a form of reality testing. Seth in no way denies the existence of an external reality:

Nothing in what I say should be taken to deny the existence of things in the world, be they onrushing trains or cats or coffee cups. The “control” in controlled hallucination is just as important as the “hallucination”. (Seth, 2021, p. 93)

Yet some writers have taken Seth’s theoretical approach to involve a general skepticism about perceptual experience:

it is hard to understand how one can motivate a general scepticism about perceptual experience on the basis of neuroscientific findings, since the latter – to some extent at least – presuppose the validity of the former… How do we at all know that there really is a brain? If we are to consider the deliverances of the senses as nothing but brain-generated constructions, that must also go for whatever they tell us about the brain. Neo-neo-Kantians like Seth need a real brain in order to qualify the world of experience as a brain-generated fantasy, but according to their own framework they are not entitled to posit the brain as real and mind-independent. If their theory is to be consistent, the brain itself must be a brain-generated construct. But if so, the account is circular, explanatorily vacuous, and self-undermining. (Zahavi, 2024, p. 213)

It is true that Seth sometimes speak about the external world as a “neuronal fantasy”, so it is understandable that Zahavi and other readers may picture him as a kind of idealist philosopher. But in other passages it is clear that Seth in no way considers our perceptions to be just “brain-generated constructions”, as Zahavi would have it. On the contrary, perception is seen as a process where brain-generated constructions are continually *tested* and *updated* by means of “the deliverance of the senses”.

This is made abundantly clear in Seth’s discussion of “precision weighting”, where the brain is said to engage in inferences about the relative reliability of sensory signals:

the relative reliability of sensory signals determines the extent to which perceptual inferences are updated. Your initial glance at a far-away gorilla… will deliver sensory signals with low reliability… sensory data with low estimated precision have weaker effects on updating prior beliefs… the precision of sensory signals is not something that is directly given to the perceiving brain. It also has to be inferred. The brain is faced not only with the challenge of figuring out the most likely causes of its sensory inputs, but also with figuring out how reliable the relevant sensory inputs are. (Seth, 2021, p.108)

This process involves attention. If we, after an initial glance at a distance, perceive something that looks like a gorilla, but we still doubt that this is the case, this means that we “down-weight” the precision of our sensory impressions. But it also means that we can increase the precision of the sensory impressions by attending in more detail to what is there to see.

As Seth describes it,

… we are all intimately familiar with the role of precision weighting in perception. Increasing the estimated precision of sensory signals is nothing other than ‘paying attention’. When you pay attention to something – for example, really trying to see whether a gorilla is out there in the distance – your brain is increasing the precision weighting on the corresponding sensory signals, which is equivalent to increasing their estimated reliability (Seth, 2021, p. 109)

Although Seth’s theory of perception as “controlled hallucination” may seem somewhat “exotic”, it brings to light aspects of the perceptual process that are largely ignored in many other theoretical approaches. Of special importance is the understanding of the “generative models” that “determine the repertoire of perceivable things”.

Other writers have also pointed to the need for theoretical concepts that can capture the natures of such internal structures or systems, although with other terms, such as “schemata” (e.g., Neisser, 1976; Piaget, 1967), “personal constructs” (Kelly, 1955), “schematic models” (Teasdale & Barnard, 1993), and “meaning structures (Lundh, 1983, 1995). The Copernican revolution that Seth has in mind probably must wait for a deeper understanding of the nature of these mind/brain structures/systems.

Two critical points that I think are worth discussing in relation to Seth’s theoretical approach are (1) his *reduction* of conscious contents to nothing more than the brain’s “top-down predictions”, and (2) his rather *passive* view of the perceptual process.

### Seth’s reductionism

There is a strange reductionism in Seth’s claim that our conscious contents are *nothing more than* the brain’s top-down predictions:

top-down predictions do not merely bias our perception. They *are* what we perceive. Our perceptual world alive with colours, shapes, and sounds is nothing more and nothing less than our brain’s best guess of the hidden causes of its colourless, shapeless, and soundless sensory inputs. (Seth, 2021, p. 115)

This seems to reduce our conscious contents to be only about the perceptual *world*. But our conscious experience also includes *feelings* of uncertainty, surprise, confusion, etc., in connection with the perceptual *process*. Here Seth reduces conscious experience to *the resulting interpretations* of what causes our sensory inputs, while ignoring the *process* leading up to these interpretations.

For some strange reason, Seth also denies that we have any conscious experience of our sensations:

We never experience sensory signals themselves, we only ever experience interpretations of them. (Seth, 2021, p. 83)

Although from a different theoretical perspective, Gibson (1966) also claims that perception is about the *world* and does *not* essentially involve any conscious awareness of sensations. However, although Gibson denies that visual sensations are important for our perception of the surrounding world, he admits that visual sensations are important for our conscious experience *of ourselves*. This is expressed in his distinction between the *visual world* and the *visual field*:

The temporary array of perspective appearances of the world is called the field of visual sensations, or the visual field, and this, I think, is the best index an observer has of himself as *here*. So I have to admit that the study of sensations is important for an understanding of one’s awareness of the self even when I deny that it is basic to an understanding of one’s awareness of the world. (Gibson, 1969, p. 409)

A main point here is that, as human beings, we are not only interested in interpreting the world, but also in how the world appears to us, and how it *feels* to be an embodied being in a surrounding world. This is an important aspect of conscious experience, which may even be cultivated for its own sake:

For some purposes we even *need* to pay attention to how the surrounding environment appears to us from our specific perspective. Painters and photographers, for example, may need to train this kind of attention. More generally, whichever sensory modality is used, it is possible to shift attention from a focus on the world around us to our perceptual *perspective* on the world. Although the body with its senses is most often a *medium* through which we perceive the world, we can also shift attention to this medium by focusing on how the world appears to us through our senses. (Lundh & Foster, 2025, p. 9)

In this perspective, Seth’s claims that “top-down predictions are what we perceive” and that “we never experience sensory signals themselves” seem difficult to defend.

### The passive view of the perceptual process

Seth puts his predictive processing theory in contrast to other information processing approaches, by emphasizing more of top-bottom than bottom-up processes. But like other information processing theories, his theory stays *within* the brain. Although he emphasizes the importance of mental *activity*, it is still only *mental* activity that is in focus. There is very little about human beings and other living beings as *actively* exploring and engaging with their environment.

Seth refers to Friston’s concept of *active inference*, but only in connection with his discussion of the regulation of internal physiological processes. The concept of active inference, however, points in the direction of another kind of perceptual theory, where the person’s *activity* has a much larger role. As expressed by Parr et al. (2022; see also Lundh, 2026):

To illustrate the simplicity of Active Inference—and what we are trying to explain—place your fingertips gently on your leg. Keep them there motionless for a second or two. Now, does your leg feel rough or smooth? If you had to move your fingers to evince a feeling of roughness or smoothness, you have discovered a fundament of Active Inference. To feel is to palpate. To see is to look. To hear is to listen. (Parr et al, 2022, p. vii)

The main point here is the importance of *motion* in the perceptual process: to perceive, we need to *move* our hands, eyes, head, and body. This is a kind of activity that is central to Gibson’s (1966) theory. At the core of perception is our ability to *actively explore* the world around us by moving our eyes and our head, by walking around in our surroundings, etc., so that the changing perspectives on things can inform us about the world around us.

One of Gibson’s (1966, 1979) main contributions to the psychology of visual perception is his demonstration of the rich information that is available in the pattern of light that is reflected from each structured environment – ecological optics. The light converges in potential observation points in the surrounding medium (air or water) that may be sampled by living individuals who move around in that environment.

Seth´s way of speaking about sense impressions in terms of sensory “signals” illustrates his view of perception as a passive process. This is in stark contrast to how Gibson (1966) sees it,

The active senses cannot be simply the initiators of *signals* in nerve fibers or *messages* to the brain; instead they are analogous to tentacles and feelers. And the function of the brain when looped with its perceptual organs is not to decode signals, nor to interpret messages, nor to accept images. These old analogies no longer apply. The function of the brain is not even to *organize* the sensory input or to *process* the data, in modern terminology. The perceptual systems, including the nerve centers at various levels up to the brain, are ways of seeking and extracting information about the environment from the flowing array of ambient energy. (Gibson, 1966, p. 5)

What is required for perception in Gibson’s view is a perceptual system that is *attuned* to the existing stimulus information and can *resonate* to it. On this view, the explanation

should be sought in the neural loops of an active perceptual system that includes the adjustments of the perceptual organ. Instead of supposing that the brain constructs or computes the objective information from a kaleidoscopic inflow of sensations, we may suppose that the orienting of the organs of perception is governed by the brain so that the whole system of input and output resonates to the external information. (Gibson, 1966, p. 5)

As Gibson (1966, p 271) puts it, “a system ‘hunts’ until it achieves clarity”, a clarity that consists in *resonance* of the system to the incoming information. From this theoretical perspective we might speak about *dis-attunement* (see also Bruineberg et al., 2018) rather than “prediction errors” when an individual encounters something new and unexpected that he or she has no established routines for handling. Importantly, this moves the basic process out of the brain into a more holistic perspective on the living individual’s interaction with their environment.

## Conscious Self

The third part of Seth’s book is about the conscious self. Here he claims that self-perception is not primarily about self-*knowledge* but about *control* and *regulation*:

We do not perceive ourselves in order to know ourselves, we perceive ourselves in order to control ourselves. (Seth, 2021, p. 170)

Self-perception is not about discovering what’s out there in the world, or in here, in the body. It’s about physiological control and regulation (Seth, 2021, p. 171).

One basic form of self-regulation is the regulation of *essential variables*, such as “body temperature, sugar levels, oxygen levels and the like, that must be kept within certain rather strict limits in order for an organism to remain alive” (p. 184). Importantly, the top-down process here is not only about *prediction* but also about *goal-directed action*. In temperature regulation, for example, the

goal is to *regulate* this inferred hidden cause, to take *action* so as to keep the temperature within a comfortable range, and ideally at a single fixed value. Perception, in this context, is not for figuring out what’s there, it’s for control and regulation. (Seth, 2021, p. 183)

It becomes difficult to follow Seth, however, when he wants to reduce all kinds of emotions and moods to conditional predictions about essential *physiological* variables:

The control-oriented perceptions that underpin emotions and moods are all about predicting the consequences of actions for keeping the body’s essential variables where they belong… the form and quality of my emotional experience are the way they are – desolate, hopeful, panicky, calm – because of the conditional predictions my brain is making about how different actions might impact my current and future physiological condition. (p. 187)

But are all emotions really about the regulation of *physiological* conditions? What about pride, shame, and guilt, for example? Various researchers (e.g., Leary, 2012) have argued that these emotions serve to regulate our s*elf-esteem* and *interpersonal relations*, rather than physiological conditions. But there is very little about such things in Seth’s book.

On a more positive note, Seth does present a rather integrative view of the conscious self, describing it in terms of various *layers*. The most basic layer is the experience of *being a body,* which Seth describes in terms of the “feeling of being alive” (p. 151). Above that, we find the experience of body-ownership (i.e., *having* a body), the *first-person perspective* (i.e., perceiving the world from a particular point of view, defined by one’s body), and the *volitional self* (e.g., the experience of agency). Still higher up, or closest to the “surface”, are the *narrative self* with “a personalized prior history, a thread of autobiographic memories, a remembered past and a projected future” (p. 152) and the *social self,* which includes “how I perceive others perceiving me” (p. 153).

I also found Seth’s writings on the volitional self quite interesting. On page 218-219 he discusses experiences such as “the feeling that I am doing what I want”, “the feeling that I could have done otherwise”, and the feeling that voluntary action “comes from within”. Seth regards the experience of exercising a kind of *causal power* in our voluntary actions as analogous to other perceptions – that is, as representing yet another example of “controlled hallucination”. Importantly, this at the same time means that the experience of causal power is no more illusory than other perceptions:

We project causal power into our experiences of volition in just the same way that we project redness into our perception of surfaces. Knowing that this projection is going on – to channel Wittgenstein one more time – both changes everything and leaves everything the same. (Seth, 2021, p. 223)

Just like other perceptions, our experiences of volition are also crucial to our survival and well-being:

Experiences of volition are not only real, they are indispensable to our survival. (Seth, 2021, p. 223)

As Seth puts it, the “reality of this kind of free will is underlined by the fact that it cannot be taken for granted” (p. 222). In other words, the fact that it is a capacity that we may *lose*, for example due to brain damage, means that it is *real*:

free will is not illusory at all. So long as we have relatively undamaged brains and relatively normal upbringings, each of us has a very real capacity to execute and to inhibit voluntary action, thanks to our brain’s ability to control our many degrees of freedom. (Seth, 2021, p. 222)

Seth describes volition as a *competence*, or capacity, that comes in degrees, and is not restricted only to humans:

there does seem to be something distinctively human about our ability to deal with complex and changeable environments through flexible, voluntary behaviour. However, the ability to exercise free will might come in degrees not only among us humans, but more widely among the animals we share our world with.” (Seth, 2021, p. 224)

Volition is probably most likely to be activated in situations where we experience uncertainty about how to act. This can be seen in contrast to situations when everything *flows* spontaneously in accordance with our various skills:

When people talk about being ‘in the moment’ or in a ‘state of flow’ – when deeply immersed in an activity they have extensively practiced – the phenomenology of volition may be entirely absent. (Seth, 2021, p. 221)

In other words, what seems to be lacking in these situations of flow is the experience of a *volitional self*. And yet, there seems to be no reason to doubt that there are “top-down processes” as well as “bottom-up processes” still going on here. To me, this suggests a perspective on consciousness not as associated primarily with top-down processes but rather with prediction errors and the work to handle these.

## Conclusion

There is much “food for thought” in Seth’s book. Still, it leaves me with quite mixed feelings. On a positive note, I welcome Seth’s openness to phenomenology in the form of conscious *phenomena*. On a more negative note, the phenomenological ambitions sometimes seem to almost “drown” in the reductionism of the predictive processing paradigm. For several reasons, as described above, I am not convinced by Seth’s theory of consciousness. But I do think he makes an interesting point when he argues for the need of a Copernican revolution in this field.


*Lars-Gunnar Lundh*


## Open access

This article is distributed under the terms of the Creative Commons Attribution 4.0 International License (http://creativecommons.org/licenses/by/4.0/), which permits unrestricted use, distribution, and reproduction in any medium, provided you give appropriate credit to the original author(s) and the source, provide a link to the Creative Commons license, and indicate if changes were made.
